# “I am not alone”. A qualitative feasibility study of eating disorders prevention groups for young females with type 1 diabetes

**DOI:** 10.1186/s40337-023-00767-2

**Published:** 2023-03-20

**Authors:** Trine Wiig Hage, Jan-Vegard Nilsen, Katrine M. Karlsen, Martine H. Lyslid, Anne Louise Wennersberg, Line Wisting

**Affiliations:** 1 Regional Department for Eating Disorders, Division of Mental Health and Addiction, Oslo University Hospital, Nydalen, P.O. Box 4956, 0424 Oslo, Norway; 2 Oslo Diabetes Centre, Oslo, Norway

**Keywords:** Feeding and eating disorders, Diabetes mellitus, type 1, Preventive programs, Body image, Body dissatisfaction, Health psychology, Population at risk, Thematic analysis

## Abstract

**Objective:**

The overall aim of the current study was to qualitatively explore the feasibility of eating disorder prevention groups for people with type 1 diabetes (T1D).

**Method:**

A generic qualitative focus group design was applied. 17 participants accepted the invitation to attend focus group interviews after completing the intervention. Five focus groups were conducted in total.

**Results:**

The qualitative analysis generated one overarching theme, named the benefit of meeting peers with a lived experience of T1D and body image concerns, and four themes: the need for an integrated focus on diabetes, personal relevance, providing sufficient balance between structure and flexibility and enabling a different perspective.

**Conclusion:**

Results show overall positive feedback regarding the content and structure of the intervention, and underline the importance of targeting preventive efforts to specific risk groups.

## Abstract (summary)

Young females with type 1 Diabetes constitute a high-risk group for developing eating problems and eating disorders. Interventions specifically targeted at preventing body image and body dissatisfaction issues in this group are therefore very important. The current study thus aimed to explore participants’ experiences with a Diabetes specific version of the targeted prevention program the Body Project, Diabetes Body Project. Participants were invited to attend focus groups interviews after completing the intervention. Results from the study consist of one overarching theme and four themes. Participants highly appreciated the opportunity to meet other young females with type 1 Diabetes, underlining the importance of interventions specifically targeted at this high-risk group. They also emphasized that the script would benefit from a more integrated focus on type 1 Diabetes throughout all six sessions of the intervention.

## Introduction

Type 1diabetes (T1D) is autoimmune, chronic disease characterized by a lack of insulin due to an autoimmune destruction of the insulin-producing beta cells in the pancreas. Lack of insulin leads to hyperglycemia (i.e. high blood glucose levels), which is associated with increased rates of diabetes complications, including neuropathy, retinopathy, and nephropathy. In addition, hypoglycemia (i.e. low blood glucose levels) may lead to acute complications such as seizure or loss of consciousness. Treatment is largely based on self-care, and include administering insulin via an insulin pen or pump. Making decisions about insulin dosing according to blood glucose values and carbohydrate portions. The treatment requires a focus on type and amount of food consumed and carbohydrate counting. This has been suggested as one aspect of T1D management that places people at greater risk for developing an eating disorder (ED) [1]. Young females with T1D constitute a specific risk-group for developing EDs [6], with prevalence rates of diagnosable EDs being 2–3 times higher than for non-diabetic peers (Colton et al., 2015, Young, 2013). The prevalence of sub-threshold eating problems, commonly referred to as disturbed eating behaviors (DEB) is even higher [17, 28]. Thus, females who have T1D and are in their late adolescence or early adulthood appear to be at particular risk for developing DEB and EDs [28]. Both EDs and DEB are associated with significant stress and impairment of daily functioning [20].

Risk factors for developing EDs, in addition to being female and of young age, include pursuit of the thin beauty ideal, body dissatisfaction, and negative affect [21]. Additionally, potential T1D specific risk factors for developing EDs include weight loss at disease onset followed by weight gain caused by insulin treatment, dietary control as part of diabetes management, and intentional insulin omission to control weight [18]. Presence of comorbid T1D and DEB/EDs are associated with poor glycemic control and increased rates of morbidity and mortality [16]. Despite the frequency and severity of this comorbidity, no studies have previously investigated effective prevention approaches targeted at this specific high-risk group.

The “Body Project” is a targeted, manualized and interactive group based prevention program, based on cognitive dissonance theory. The Body Project has, through a number of studies, been found to be the most effective intervention to reduce ED risk factors and symptoms [13, 26], and to prevent future ED onset among young females in the general population [21]. According to Festinger [7], cognitive dissonance occurs when there is a discrepancy between one’s beliefs and one’s actions. This inconsistency is theorized to create psychological discomfort, which then motivates the individual to reduce the cognitive discord by changing its beliefs. In Body Project, participants are encouraged to criticize the thin ideal through a series of verbal, written, and behavioral exercises, including homework assignments. These activities are designed to produce cognitive dissonance, thus reducing internalization of the thin body ideal, which in turn is thought to result in reductions in body dissatisfaction and ED symptoms. A qualitative study investigating experiences with the original four-session Body Project manual [19] found that participants reported an overall satisfaction with the intervention, with the group´s format being viewed as most valuable, followed by the opportunity to discuss issues with the other participants in a group setting. The program´s scripted nature was noted as less valuable by participants. Originally, Body Project was designed as a face-to face intervention. Ghaderi et al. [8] developed and tested a virtual version of the intervention. Their results suggest that a virtual delivery of Body Project groups may be an effective and accessible prevention strategy for individuals at risk for EDs.

Given the robust evidence of the effectiveness of the Body Project for individuals without T1D, Wisting et al., [27] developed a virtual, T1D specific version of the Body Project, Diabetes Body Project, and tested the feasibility and acceptability of that script. Results from the quantitative part of the study suggest that the Diabetes Body Project is a feasible and acceptable ED prevention approach for females with co-occurring T1D and body image concerns. Moreover, statistically significant within-subject reductions in ED risk factors and symptoms from baseline to posttest were reported. In the current article, we report qualitative feasibility undertaken as part of the pilot study. The use of qualitative research is specifically recommended in assessing whether an intervention engages a distinct target group [5].

The overall aim of the current study was to investigate the feasibility, using qualitative methodology, of the adapted Body Project for individuals with type 1 diabetes. Specifically we aimed to (a) explore participants’ experiences with the practical organization and the way the group intervention was conducted, (b) explore participants’ experiences with the content of the intervention and (c) explore the potential impact that taking part in the program had on participants´ daily life. The results of this study, together with the quantitative results previously reported, may lead to refinements of the script and inform the development of future trials.

## Methods

This paper is part of a larger project focusing on the development and piloting of Diabetes Body Project [27]. The project was supported by Dam Foundation, conducted in collaboration with the Norwegian Diabetes Association and led by the Regional Department for Eating Disorders, division of Mental Health and Addiction, Oslo University Hospital.

### Design

A generic qualitative focus group design was applied, which aimed to generate a comprehensive summary of the key themes emerging from the focus group discussions [2, 11]. Focus group interviews are recognized as an effective and suitable method for exploring specific topics. In addition, the focus on discussions and interaction between group participants may generate new questions and highlight important aspects the researcher is unable to anticipate beforehand (ibid).

### Diabetes body project group intervention

In the development of Diabetes Body Project, the latest version of the original four-session Body Project script [22] was translated to Norwegian. In the first four sessions, participants are asked to define, discuss and challenge current appearance ideals, as well as engage in body activism activities and discuss advantages and disadvantages of social media use. Examples of verbal and written in-group exercises and homework exercises include a variety of letter writing exercises and the “mirror exercise”, a homework assignment where participants are encouraged to find positive attributes about themselves, including at least two on appearance. The format and the content of the groups are designed to maximize cognitive dissonance. The script was adapted to include issues specifically related to T1D by adding two diabetes specific sessions following the original four sessions, delivered in the same interactive format. Specific details of the content of the six-hour Diabetes Body Project is described in the initial report [27].Two to individuals with lived experience with T1D (KMK & MHL) contributed to the development of the diabetes-specific content. Examples of T1D specific content include group discussions concerning costs of not managing the illness appropriately as well as costs of misusing insulin for weight management. Thus, the Diabetes Body Project feasibility study [27] involved six weekly delivered 1- hour sessions. Inspired by Ghaderi et al. [8], a virtual-delivery format was applied.

### Participants and procedure

35 female participants were included in the Diabetes Body Project feasibility study, and allocated to five Diabetes Body Project Groups. 26 participants completed all six meetings, including pre- and post-tests (delivered online). Mean age of onset of T1D was 9.34 (SD 6.03). Mean total score on the Diabetes Eating Problem survey-revised (DEPS-R) was 18.4 (*SD* 9.03). At baseline, 42.9% of the participants scored above the DEPS-R cut-off (≥ 20), versus 26.9% at posttest. Mean age of participants was 25.62 (3.92). 9 participants chose to drop out during the course of the groups. These participants did not differ significantly from participants completing all sessions in terms of baseline demographic and clinical characteristics. For a more detailed description of recruitment and sample procedures, see Wisting et al., [27]. All participants who completed the Diabetes Body Project group intervention were invited to participate in focus group interviews after program completion. A total of 17 out of the 26 participants accepted the invitation. Mean age of focus group participants was 26.61. Participants who declined the invitation to participate in the focus groups were either sick or otherwise engaged, e.g. with school exams. Five focus groups were conducted in total, with between 2 and 4 participants in each group. Both the Diabetes Body Project intervention and the focus group interviews were carried out during a phase of the Covid-19 pandemic characterized by lockdown.

The focus group interviews were conducted on the same virtual platform as the Diabetes Body Project intervention and lasted between 53 and 71 min. Participants confirmed they were alone at the beginning of the interview. The same procedure was applied for each Body Project session. All five groups were moderated by a researcher with competence in conducting qualitative research (TWH and JVN), and a co-facilitator (ALW, KMK and MHL). Two of the facilitators (AL and TWH) had participated in running some of the intervention groups. In order to limit potential biases due to social desirability concerns, they did not conduct focus group interviews with these groups.

A semi-structured interview guide was developed for the purpose of the study (available upon request). The guide was iteratively developed based on feedback from the two individuals with lived experience with T1D (KMK and MHL) and the Body Project team at the Regional Department for Eating Disorders at Oslo University Hospital. The guide was structured around two main points: overall experiences with group participation and questions concerning the content and format of the group intervention. Questions were open-ended. In order to enhance data richness, facilitators were encouraged to probe participant responses. All interviews were audio recorded and transcribed verbatim. Ethical approval for the study was obtained from the regional ethical committee (REC) north (reference 6860). All participants signed informed consent forms.

### Data analysis

The audio recordings were transcribed verbatim by a professional transcription service and entered into NVivo (version 10) for initial organization and coding. Parts of the analysis was also conducted manually. Braun and Clarke’s [3] six steps of reflexive thematic analysis were used to analyze the qualitative data. The steps included (1) familiarization with the data, (2) generation of initial codes, (3) search for themes, (4) review of themes, (5) name and definition of themes connected to focus areas, and (6) report creation. The initial long list of themes and subthemes created from the data was reduced through comparison across the different focus groups and participants within the groups, guided by the research aims. The initial analysis was conducted by TWH and JVN. In line with Braune and Clarke [4], the coding approach when more than one researcher is involved was collaborative and reflexive. Embedded in this approach, is the acknowledgement that knowledge is never free of researcher influence, that our assumptions, values and choices shape the knowledge we create. An essential part of the analytic process was thus a critical reflection of our roles as researchers, designed to develop a richer and more nuanced interpretation of the data, rather than seeking a consensus on meaning. After completing the initial analysis, the two user representatives met with JVN and TWH and discussed the overall impression and tentative themes from the interviews. The two individuals with experience with T1D (KMK & MHL) were invited to share their overall impressions based on their experience with co-moderating some of the focus groups, before they were presented with the tentative themes. Although their feedback largely resonated with the preliminary thematic structure, it also brought new perspectives and insights into the data material, thus providing a broader and deeper understanding of the data.

## Results

The qualitative analysis generated one overarching theme, named “the benefit of meeting peers with a lived experience of T1D and body image concerns”, and four themes; *the need for an integrated focus on diabetes, personal relevance*, *providing sufficient balance between structure and flexibility* and *enabling a different perspective*. The relationships between the themes are illustrated in the figure below. A more detailed presentation using examples from the data corpus follows. The extracts were chosen because they contained illustrative information of the participants’ experiences and illuminate various aspects of the total data corpus. Extracts from focus group interviews were translated by TWH and JVN from Norwegian to English, as directly as possible. All names are pseudonyms. Comments in brackets in the extracts are the authors’ own explanations / interpretations.

### The benefit of meeting peers with a lived experience of T1D and body image concerns

The overarching theme, as generated by the participants in the focus groups, represents an abstraction of the main themes. Participants unanimously expressed that meeting peers with T1D overall was a highly valuable and appreciated experience. The group represented a temporary safe space that enabled a connection with a community of peers. Learning from the lived experience of peers, e.g. how fellow group members chose to solve home assignments and reflect on issues addressed throughout the groups, appeared to promote self-reflection and stimulate new perspectives regarding own attitudes and behaviors. That all participants had a lived experience of T1D seemed to strengthen the feeling of being in this together. Many participants described a limited network of other females with T1D, and thus particularly valued meeting peers. This seemed true for both participants relatively recently diagnosed with the disease and for those who had lived with T1D for most of their lives. Across all groups, most participants shared positive experiences with the virtual group format. Being able to attend the meeting from home was both described as convenient and safe, even when discussing and sharing personal issues.

### The need for an integrated focus on T1D

In four of five focus groups, participants expressed that the clear division between general issues pertaining the thin beauty ideal in the first four sessions of the Diabetes Body Project intervention and the focus on T1D specific content in the last two sessions was experienced as artificial. *“I thought it was difficult to discuss body and exercise and food and everything without including the diabetes, that it was separated in the first sessions. It was like; “We are not addressing the diabetes now, now we are talking about the body”. At least for me, and I believe that others [participants] had the same impression, you can’t separate these things easily” (Kari, group 3)”.*

Most participants called for a greater emphasis on T1D from the outset of the intervention. Some also expressed that the program was not aligning with their expectations, as they believed they had signed up for a program emphasizing dilemmas and challenges related to living with T1D. The majority agreed that it took too long before T1D was addressed in the two last sessions of the intervention. As Siri (group 3) put it: *“They [the group leaders] had a strong agenda for what should be in focus, and I found that a bit peculiar, as this [diabetes] was my reason for signing up”.*

Although these views were strongly highlighted in a majority of the focus groups, some participants also emphasized that they appreciated the generic focus on the thin ideal and body image issues, as well as the T1D specific content. Thus, Anne (group 2) also stated that the diabetes focus needs to be well balanced too, as* “We are not just diabetics, we are normal young females living in a society as all others, with all that holds.”.*

Across all focus groups, participants expressed that living with T1D represented specific challenges related to health behaviors and body image, and the T1D specific content in the Diabetes Body Project intervention was highlighted as particularly helpful. Several individuals underlined that diabetes-specific challenges were experienced as highly useful to discuss with peers, like significant scar tissue from insulin injections, the insecurities of using visible equipment like glucose monitoring sensors or insulin pumps, or difficulties with having to eat when not hungry, in order to treat or prevent hypoglycemia.

“I think it was a very good thing that we were able to talk about the body and the challenges from a standpoint of [being] female with diabetes, because I experience that these conversations are very different than they are with others without diabetes” (Caroline, group 5).

### Personal relevance

Although most participants seemed to enjoy discussions of the thin ideal and other aspects of living in a contemporary culture promoting specific body ideals, quite a few of the participants reflected on whether the program’s focus was too narrow to allow for personal relevance. Aligning with this, several experienced that they currently were not part of a social context where the thin ideal or strict body ideals were common, leaving them with a feeling that the program’s main focus was somewhat off target. As Marie (group 1) put it:

“*For me and many others it is about, not necessarily to look thin, as an ideal, but it can be muscular or fit or that you look a certain way. So exactly the “thin ideal”, I do not know if the ideal always is thin. When we spoke of the “beauty ideal”, then I felt it was a more inclusive concept, that could refer to both looks, how you look, your appearance, the body, it wasn’t just about being thin”.*

Overall, participants across all groups called for a broader focus when addressing contemporary culture and several spoke of the importance of the program allowing sufficient flexibility to ensure personal relevance for each participant, both regarding content and homework assignments. Most participants thought that an integrated focus on aspects concerning the body or body image, different health behaviors, self-esteem, and the unique experiences of living with T1D could have increased the feeling of personal relevance. For some, the focus on social media use, both during the sessions and as part of homework assignments, was experienced as irrelevant, because they rarely posted on social media sites. Some also described that much of the content in the sessions was aimed at a younger age group, and was thus experienced as less relevant for the participants in the older age range (< 20).

*“Sometimes I felt that it was as if you were aiming for certain difficulties that I don’t experience”* (Caroline, group 5).

Importantly, most participants reflecting on the potential behavior changes that could be attributed to taking part in the sessions, emphasized that such behavioral changes usually occur in social settings not permitted during an ongoing pandemic (Covid-19 pandemic). Moreover, participants in the higher end of the age range said that several of the interventions and tasks would probably have been more beneficial/useful a few years earlier, because insecurities due to or concerning body image issues had diminished with age.*”I felt, when I joined this group, that I am a bit past having a difficult relationship with food and (my) body. I have been there, but I am not there now. So I am kind of thinking: «oh, this should have been a few years back» (Pia, group 4).*

### Providing sufficient balance between structure and flexibility

The structured group format seemed to enable sufficient security for participants to share personal information that they would otherwise not be likely to share in their daily life. As Veronica reflected: “…*then I heard that the other participants spoke of experiences similar to mine, and then I dared to talk about my experiences [too] and reflect on these”* (Veronica, group 3).

Many participants in several of the focus groups reflected on the set duration of the Diabetes Body Project intervention. Many described 1 h as a good length, making it possible to attend despite a busy life schedule.

*“What I like with an hour, is that it is totally doable for me to set aside an hour and take part. If it was longer, it might be, like, that it would take up too much of my time” (Anne, group 2).*

Some participants thought that the duration of the intervention could have been more flexible, allowing for more interaction and discussion between participants. Due to the scripted nature of the manual, the sessions sometimes were experienced as rushed, and it was necessary to move on and complete all of the scheduled exercises.

*Much of the benefit lies in being able to respond and start a conversation based on somebody’s answer. And that I felt was very difficult to do, because you had to move on, all the time. If you started to say something after somebody had read their home assignment, it was: Ok, but now we have to move on […] they did not give us room to talk, that was not part of the framework (Linn, group 4).*

Participants suggested that a possible solution to this was to have longer sessions or a more flexible use of the manual, allowing for more discussions during some of the exercises, and making the manual a better fit with the dynamics in each group and the individual needs of each group member. For example, Sarah (group 3) rhetorically asked: “*Did we have to listen to all homework assignments, every session?* “Others suggested that if one hour is set as the program’s time frame one could think about the possibility of inviting participants to optionally stay for another thirty minutes together with group leaders to discuss further.

### Enabling a different perspective

As a whole, participating in a group format encouraging the exchange of thoughts, feelings and experiences associated with the different topics addressed in the group sessions, was valued as predominantly beneficial by participants across all groups.

Several of the focus group discussions revolved around participants experiencing an enhanced self-awareness related to the different themes discussed throughout the sessions and by engaging in the homework assignments. Across all groups, participants reported, in one way or another, that group participation had stimulated their self-reflexivity concerning own body image and how the thin beauty ideal and/or different societal pressures both had and presently affected them. Throughout the focus group discussions, participants described both an enhanced awareness of their “inner voice”, i.e. how they talked to or of themselves, and a renewed awareness of how they verbally communicated with others. As described by Siri (group 3), participation in the Diabetes Body Project group enabled her to “*increase [my] awareness on the difference between the thin ideal and what is health promoting [for me]*”.

Several of the participants had experienced, during the course of group participation, an increased attentiveness to their own language use and reported a new interest in how choice of words could come to influence both themselves and others, in potentially both positive and negative ways. This reflection had also led some to become more aware of their own responsibilities while engaging in conversations regarding the body, cultural ideals and different health behaviors with friends, family and colleagues.

“I *have in fact corrected a couple of friends, maybe with more confidence than before… and this led to good discussions (with my friends) that I felt was surprising” (Sophie, group 3)*.

Being able to listen to the other participants during group discussions and witnessing how they had solved the home exercises were viewed as the most useful components of the intervention. Listening to and observing others seemed to stimulate self-reflexivity, as different perspectives on the same subjects emerged. Exercises that were highlighted as particularly beneficial for enhancing self-awareness and countering societal pressures were the “mirror exercise” (i.e. standing in front of the mirror and recording positive aspects of themselves, including physical), role-play exercises within the session where group leaders acted as people obsessed with the thin beauty ideal, and variations of letter writing. *«I thought that the mirror exercise was both nice, but also a bit challenging. It really made me think. It really evoked emotions. Nice emotions too» (Kari, group 5).*

Moreover, an increased awareness of the influence of social media on body image, as well as personal social media use, was mentioned by participants across all groups.

*“(..) Things like: who you follow on social media, what you give a “like”. Things like that. How much you actually contribute to this body image pressure. If you like people who stand for (that ideal) or support businesses who do, you are part of maintaining it. So I feel that I have become a lot more conscious after we have talked (about it)” (Sophie, group 3).*

## Discussion

Findings from the current study yield important information about young females’ with T1D experiences with participating in Diabetes Body Project groups. Results emphasize the value of this targeted and interactive prevention program, as well as pinpointing some specific challenges and potential areas for improvement of the Diabetes Body Project script. The overarching theme underlined the benefit of meeting peers with T1D and body image concern. This is in line with previous research investigating participants experiences with the original four session Body Project intervention, which have reported that a sense of belonging and not feeling alone with their body dissatisfaction are experienced as the most important aspects of participants’ experiences [10, 19, 25]. Recognizing shared experiences, group cohesiveness, and developing hope from seeing others change are commonly reported benefits of psychotherapeutic group interventions [29]. Moreover, the Diabetes Body Project likely represents the first time several of the participants had the opportunity to voice and discuss their body image concerns with other young females with T1D. For this group, discussing and interacting with peers can be particularly important due to illness specific challenges experienced during early adulthood, in addition to normal challenges for this age group. Young individuals with T1D have previously described that other individuals with T1D are the only ones who can truly recognize how living with this disease feels and what it entails [9]. Moreover, loneliness has been reported to be more frequent among people with diabetes, and it seems that this was enhanced during the Covid 19 pandemic [14], thus underlining the need the need for opportunities to socialize and connect with peers with T1D.

Participants highlighted the need for a more integrated focus on T1D throughout the Diabetes Body Project intervention. Findings from qualitative studies exploring young females experiences of living with T1D, emphasizes that integrating and accepting T1D as part of participants’ identity is central when it comes to accepting the illness as well as living a normal life [12]. Thus, although we have kept the original division of four plus two sessions in the Diabetes Body Project script, we have we have adapted the format to include T1D topics in both in-group exercises and homework throughout all 6 group sessions..

Results from the current study indicate that the term the thin beauty ideal was experienced as a bit too narrow as it did not cover all experienced issues related to being exposed to the current beauty ideal. In recent years it appears to be a shift in the body type to which women aspire from the traditional thin ideal toward being fit and toned (i.e., the fit ideal) or the thin – thick body ideal, with a flat stomach and larger thighs and butt [15]. We have incorporated this into the Diabetes Body Project script by using the terms thin ideal, beauty ideal and appearance ideal interchangeably, thus allowing for a broader definition when describing and discussing current societal expectations to appearance.

The scripted nature of the Diabetes Body Project could be experienced as a bit inflexible by the participants in the current study, making the intervention feel a bit rushed and allowing for few lengthy discussions. This is similar to Shaw et al.’s finding in their study of participants’ experiences with the original Body Project intervention, where the program’s scripted nature was noted as less valuable and in need of improvement. Moreover, Jarman et al., [10] found that, for some participants, the scripted nature of the intervention acted as a barrier to full participation and did not allow freedom to explore, develop, and discuss their own ideas. Lack of flexibility is a common critique against manualized treatments [24]. However, as manual adherence is important to maximize cognitive dissonance [23], and the script facilitates fidelity [10], Shaw et al. [19] suggest that training of group leaders should emphasize the importance of learning the script well enough to allow a natural and dynamic delivery.

Findings demonstrate that a majority of the participants experienced both enhanced self-awareness and self-reflexivity during the course of the Diabetes Body Project intervention, thus underlining the importance of discussing and challenging current appearance ideals as well as supporting theorized mechanisms of change according to cognitive dissonance [10]. Of the specific exercises, letters, role-plays and the mirror exercise were described as most valuable. These findings are in concordance with previous studies on experiences with the general Body Project script [19, 25]. As described by participants, the nation-wide social restrictions at the time limited their opportunities for social encounters, thus possibly affecting potential effects of the intervention.

There are some limitations to this study that deserve consideration. As the data from this qualitative research was part of a feasibility study, all findings should be interpreted with caution and not assume representation of all young females with T1D. Focus group facilitators had been involved in running some of the Diabetes Body Project groups thus potentially affecting the dynamics in the focus groups as well as the gathered data Due to dropout in the intervention groups and for the focus groups interviews, 17 of the originally enrolled participants in the Diabetes Body project intervention (49%) participated in focus group interviews, possibly limiting the information gathered in the interviews. Finally, as this was a pilot study, it is not clear based on the past quantitative (ref [27] results and the results of the current paper whether the Diabetes Body Project may actually prevent ED onset. A future randomized controlled trial (RCT) is need to examine this further.

## Conclusion

The current study aimed to qualitatively investigate the feasibility Diabetes Body Project groups. Results show overall positive feedback regarding the content and structure of the intervention, and underline the importance of targeting preventive efforts to specific risk groups. Refinements of the Diabetes Body Project script include allowing for a broader definition of the current beauty ideal as well as a more integrated focus on T1D throughout the intervention.

## Data Availability

The dataset used during this study is not publicly available due to sensitivity and privacy issues, but are available from the corresponding author upon reasonable request.

## Abbreviations

ED: Eating disorder
T1D: Type 1 Diabetes
DEBS: Disturbed eating behaviors
